# Factors XI and XII as Targets for New Anticoagulants

**DOI:** 10.3389/fmed.2017.00019

**Published:** 2017-02-24

**Authors:** Jeffrey I. Weitz, James C. Fredenburgh

**Affiliations:** ^1^Department of Biochemistry and Biomedical Sciences, McMaster University, Hamilton, ON, Canada; ^2^Department of Medicine, McMaster University, Hamilton, ON, Canada; ^3^The Thrombosis and Atherosclerosis Research Institute, McMaster University, Hamilton, ON, Canada

**Keywords:** thrombosis, hemostasis, contact pathway, anticoagulant, factor XI, factor XII

## Abstract

Compared with vitamin K antagonists, the direct oral anticoagulants (DOACs) are simpler to administer and are associated with less intracranial bleeding. Nonetheless, even with the DOACs, bleeding still occurs and many patients with atrial fibrillation fail to receive anticoagulant thromboprophylaxis because of the fear of bleeding. Therefore, there is an urgent need for safer anticoagulants. Recent investigations into the biochemistry of hemostasis and thrombosis have identified new targets for development of novel anticoagulants. Using data from complementary sources, including epidemiological studies and investigations in various animal models, the contact pathway has emerged as a potential mediator of thrombosis that plays a minor part in hemostasis. Consequently, factor (F) XII of the contact system and FXI in the intrinsic pathway have been identified as potentially safer targets of anticoagulation than thrombin or FXa. However, further studies are needed to identify which is the better target for the appropriate indication. This review highlights the evidence for focusing on FXI and FXII and examines the novel approaches directed at these new targets. These emerging strategies should address current unmet medical needs and provide new avenues by which to improve anticoagulant therapy by reducing the risk of bleeding.

## Introduction

The goal of anticoagulation therapy is to attenuate thrombosis without perturbing hemostasis. Although the direct oral anticoagulants (DOACs) come closer to this goal than vitamin K antagonists, bleeding is not eliminated with the DOACs. Thus, even with the DOACs, the annual rate of major bleeding in patients with atrial fibrillation is 2–3%, while the annual rate of intracranial bleeding is 0.3–0.5% (1). Consequently, because of the fear of bleeding, over one-third of patients with atrial fibrillation fail to receive any anticoagulant prophylaxis and among those given anticoagulation therapy, up to 50% are inappropriately treated with lower doses of the DOACs (2, 3). Therefore, there remains a need for safer anticoagulants.

The DOACs inhibit factor (F) Xa or thrombin, downstream enzymes in the coagulation cascade. Interest in FXII and FXI, which are upstream to FXa and thrombin, stems from basic and epidemiological studies that suggest that these factors are important in thrombosis (4–6). This makes them promising targets for development of safer anticoagulants because FXII and FXI have little or no role in hemostasis. This paper describes the rationale and approaches to targeting FXII and FXI.

## Role of the Contact System in Thrombosis

**Figure 1:**
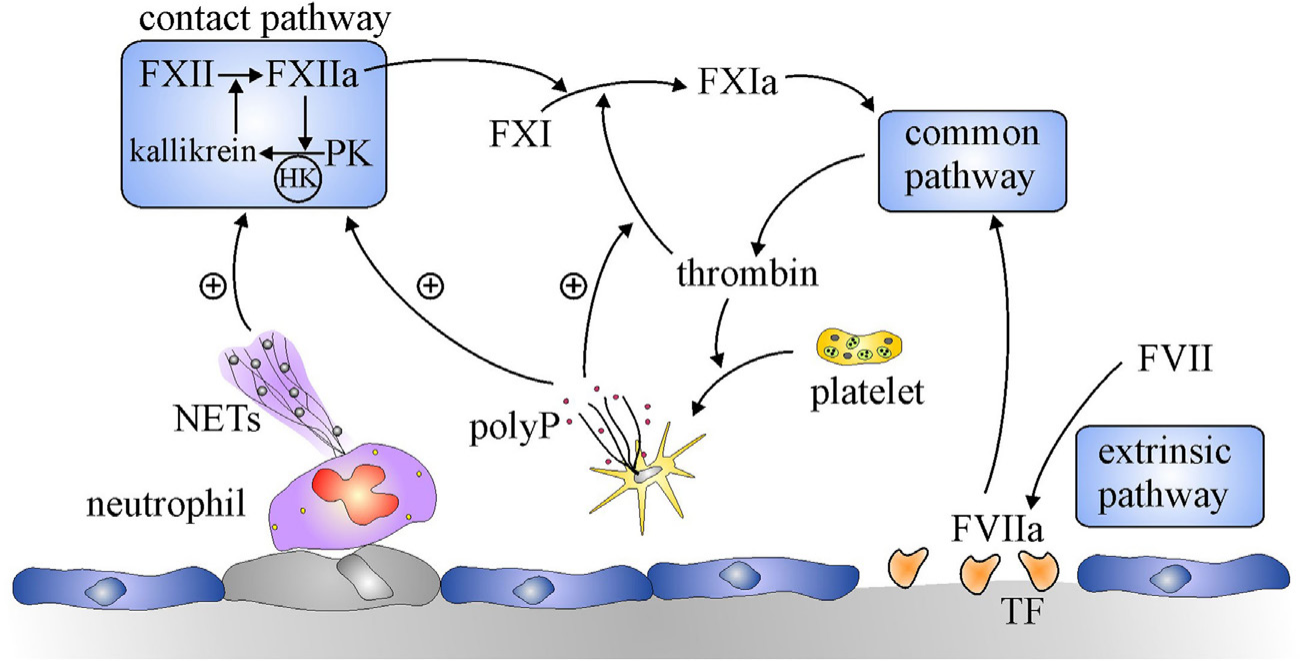
**Overview of the coagulation system**. Coagulation is initiated by the extrinsic pathway when tissue factor (TF) exposed at sites of vascular injury binds and activates factor (F) VII. The activated FVII (FVIIa)–TF complex activates FX in the common pathway to generate prothrombinase, which generates thrombin. Additional activation of coagulation occurs when thrombin-activated platelets release polyphosphate (polyP) and activated neutrophils extrude DNA and histones to form neutrophil extracellular traps (NETs). NETs and polyP activate the contact pathway, where FXII and prekallikrein (PK) reciprocally activate each other to generate FXIIa and kallikrein, respectively. The resultant FXIIa activates FXI to FXIa which leads to additional thrombin generation *via* the common pathway. PolyP amplifies this pathway by promoting thrombin-mediated activation of FXI.

Although it is dispensable for hemostasis, the contact system is essential for thrombus stabilization and growth because thrombi formed at sites of arterial or venous injury in mice deficient in FXII or FXI are small, unstable, and prone to embolization (7, 8). The contact system is composed of two zymogens, FXII and prekallikrein (PK), and a cofactor, high molecular weight kininogen (HK) (Figure 1) (6, 9). The system is initiated upon exposure of polyanionic compounds originating from injured cells or pathogens. These compounds bind FXII and HK, commencing a reciprocal activation system. Thus, FXII is activated to FXIIa through an autocatalytic reaction involving Zn^2+^, whereas HK bridges FXI and PK together to bring them into proximity of FXII. In this cyclic system, FXIIa activates HK-bound PK, and the resultant kallikrein activates additional FXII. FXIIa also activates FXI in a HK-dependent fashion, and the subsequent FXIa then feeds into the intrinsic pathway by activating FIX, leading to thrombin generation. A more recent discovery provided a missing link in regulation of the coagulation. Thus, Gailani and Broze observed that thrombin activates FXI in a positive feedback reaction (10). Although this appeared to obviate the role of the contact system by providing an alternative means of activating FXI, it nevertheless identified new levels of regulation of coagulation. Therefore, because the pathways for its activation are bidirectional, FXI is important for maximizing thrombin generation, thereby revealing an important role for the contact system.

Another reason why the contact system was overlooked for decades was that the only known activators of the contact system were artificial surfaces, such as kaolin and ellagic acid, and catheters or extracorporeal circuits, such as those used for cardiopulmonary bypass or hemodialysis (11, 12). Although physiological activators, including heparin, collagen, and denatured proteins, were known, their involvement in thrombotic disease was inconclusive (13). Renewed interest in the contact system occurred with the recent demonstration that naturally occurring polyphosphates serve as potent activators (14, 15). These polyphosphates include DNA and RNA released from injured or dying cells, inorganic polyphosphates released from activated platelets, and intact or degraded neutrophil extracellular traps (16, 17). Therefore, exposure of these activators at sites of vascular injury provides a stimulus for coagulation distinct from tissue factor (TF), identifying a potential role for the contact system in thrombosis, but relinquishing involvement in routine maintenance of blood fluidity or hemostasis (5, 18).

Coincident with description of novel physiological activators, population studies also pointed to involvement of the contact system in thrombosis. Epidemiological data support the role for FXI in thrombosis more than those for FXII. Thus, patients with congenital FXI deficiency are protected from venous thromboembolism (VTE) and ischemic stroke, subjects with higher levels of FXI are at greater risk for VTE and ischemic stroke than those with lower levels, and the levels of FXI correlate with stroke risk in women taking oral contraceptives (19, 20). The role of FXI in myocardial infarction is less clear; some studies suggest that it is important while others do not (21, 22). This discrepancy may reflect differences in study design or the contribution of FXI to thrombosis in the coronary circulation may be distinct from that in other vascular beds.

By contrast, epidemiological evidence for a role of FXII in thrombosis is not strong, but data are limited because FXII deficiency is rare (20). Patients with congenital FXII deficiency do not appear to be at lower risk for VTE, ischemic stroke, or myocardial infarction, and no differences in VTE are noted across the range of FXII levels (21). In fact, some studies suggest that such patients are at higher risk for thrombotic events. Finally, patients with hereditary angioedema as a consequence of impaired regulation of FXIIa and kallikrein due to reduced levels of C1 inhibitor or gain-of-function mutations in FXII are not prone to thrombosis. Therefore, there is little evidence of a link between FXII and thrombosis in humans.

Animal models provide a different emphasis on the roles of the contact factors since FXII-deficient mice are protected from ischemic stroke and form smaller thrombi after venous flow restriction (23). Likewise, in a rabbit model, FXII knockdown with an antisense oligonucleotide (ASO) reduced catheter thrombosis to a similar extent as FXI knockdown (24). Furthermore, mice deficient in FXII or FXI exhibit equally attenuated thrombosis at sites of arterial or venous injury, and the thrombi formed in such mice are unstable under flow conditions and undergo rapid fragmentation (7, 8). Even deficiency of PK or HK confers resistance to thrombosis in mouse injury models (21).

The results in non-human primates for FXI are similar. Thus, FXI knockdown with an ASO reduced thrombosis in a baboon arterio-venous shunt model in a concentration-dependent manner once FXI levels were below 50% of normal (25). The results for FXII are different because antibodies against FXI appear to attenuate platelet and fibrin deposition in the same model more than those directed against FXII (26, 27). Therefore, FXI appears to be a more important driver of thrombosis than FXII in non-human primate models, possibly revealing differences in regulation of FXI activation in primates versus lower mammals.

## Targeting the Contact Pathway

Experimental and epidemiological data suggest that FXI and FXII are the preferred targets in the contact pathway for development of novel anticoagulants. Targeting FXI may be associated with bleeding, particularly mucosal bleeding, which can occur in patients with severe congenital FXI deficiency (28). Conversely, because the bleeding diathesis with FXI deficiency is usually mild and variable even with the same levels of FXI, it is possible that unidentified genetic or biological factors may modulate bleeding. If so, it may be possible to compensate for them.

Factor XII is attractive as a target because of safety. Because it has no role in hemostasis and deficiency is asymptomatic, strategies targeting FXII will not induce bleeding (Table 1). A potential limitation of FXII as a target is that its role in thrombosis is less certain than that of FXI, based on epidemiological data (29–35). In addition, targeting FXII may be of limited benefit when thrombosis is initiated by TF because thrombin generated *via* extrinsic tenase has the potential to activate FXI, thereby bypassing FXII inhibition (10). Therefore, despite the potential for mild bleeding, FXI may be a better target than FXII for most indications.

**Table 1:** **Relative advantages and disadvantages of factor (F) XII or FXI as targets for new anticoagulants**.

	FXII	FXI
Epidemiological data	Weak	Strong
Risk of bleeding	None	Low
Level of evidence for role in thrombosis	Preclinical	Phase 2
Potential for bypassing inhibition	Thrombin-mediated back activation of FXI could bypass FXII inhibition	None
Potential for off-target effects	May modulate inflammation by inhibiting bradykinin generation	Low

An exception to targeting FXI may be clotting induced by medical devices or extracorporeal circuits because thrombosis on artificial surfaces is triggered by FXII activation (12). Thus, coating catheters with the potent FXIIa-directed corn trypsin inhibitor allow them to remain patent longer than uncoated catheters when inserted in the jugular veins of rabbits (36). Likewise, FXII knockdown in rabbits prolongs the time to occlusion of uncoated catheters by over twofold (24), and an FXIIa-directed antibody is as effective as heparin at preventing clotting in an extracorporeal membrane oxygenation circuit in rabbits, but produces less bleeding (37). Although FXII triggers clotting on artificial surfaces, FXI also is important. FXI knockdown is as effective as FXII knockdown at preventing catheter occlusion in rabbits (24). Furthermore, although FXII depletion reduced thrombin generation induced by components of mechanical heart valves to background levels, FXI depletion abolishes it (38). Therefore, although strategies targeting FXI may be as or more effective than those targeting FXII for prevention of clotting on artificial surfaces, targeting FXII may provide a safety benefit because of its limited role in hemostasis.

## Strategies to Inhibit FXII and FXI

With the passive roles of FXI and FXII in hemostasis, novel anticoagulant approaches with minimal risk of bleeding are being explored (4). Strategies to target FXII and FXI include (a) ASOs that reduce hepatic synthesis of the clotting proteins (24, 25, 39), (b) monoclonal antibodies that block activation or activity (24, 25, 39, 40), (c) aptamers (41), and (d) small molecules that block the active site (42–44) or induce allosteric modulation (45, 46), or agents that neutralize nucleic acids or polyphosphate as contact pathway activators (47–49). Each strategy differs not only in terms of mechanism of action, but also in mode of administration (4). Thus, ASOs, antibodies, and aptamers require parenteral administration, whereas small molecule active site inhibitors have the potential for parenteral or oral delivery. The pharmacological characteristics also vary. The 3–4 weeks of ASO treatment required to lower FXII or FXI levels into the therapeutic range limits their utility for initial treatment of established thrombosis or for immediate thromboprophylaxis (24, 25, 39). The prolonged half-life of FXI-directed antibodies or ASOs could be problematic if there is bleeding with trauma or surgery. Therefore, each strategy has strengths and weaknesses for clinical development.

## Clinical Trials

The first agent to target the contact pathway and be tested in humans is the FXI-directed ASO IONIS-416858, which is given subcutaneously and reduces FXI antigen and activity levels in a concentration-dependent manner. In a phase II study in patients undergoing elective knee replacement, 300 patients were randomized to receive subcutaneous IONIS-416858 at doses of 200 or 300 mg starting 35 days prior to surgery, or to enoxaparin at a dose of 40 mg once daily starting after surgery (50). Both treatments were continued for at least 10 days at which point patients underwent bilateral venography. The primary efficacy outcome was VTE, which included the composite of asymptomatic deep-vein thrombosis (DVT), symptomatic DVT or pulmonary embolism, and VTE-related mortality, while the principal safety outcome was the composite of major and clinically relevant non-major bleeding. In the IONIS-416858 treatment groups, mean FXI levels were reduced to 38 and 28% of baseline values in those receiving the 200 and 300 mg, respectively (50). The primary efficacy outcome occurred in 36 of 134 patients (27%) and in 3 of 71 patients (4%) who received the 200 and 300 mg doses of IONIS-416858, respectively, as compared with 21 of 69 patients (30%) who received enoxaparin. The 200 mg IONIS-416858 regimen was non-inferior and the 300 mg ASO regimen was superior to enoxaparin (*P* < 0.001). The rates of the composite of bleeding were 3% in both IONIS-416858 groups and 8% in the enoxaparin groups; differences that were not statistically significant. Therefore, lowering FXI levels reduces the risk of postoperative VTE to a greater extent than enoxaparin without significantly increasing the risk of bleeding.

In addition to providing proof of principle for targeting the contact pathway, the findings of this study change our thinking about the pathogenesis of postoperative venous thrombosis. There is little doubt that thrombin generation is increased at the surgical site as a result of TF exposure. However, the origin of this thrombin appears to also involve upstream factors. Thus, TF-induced thrombin generation may amplify coagulation by feedback activation of FXI. In addition, surgery may trigger the release of DNA and RNA from damaged cells and polyphosphate from activated platelets that directly activate FXII. These possibilities illustrate the mechanistic differences of targeting FXI versus FXII. Knockdown of FXI prevents propagation of coagulation by either pathway, whereas strategies that target FXII only block contact activation. We now need to widen the search for potential clinical indications and identify the optimal target for FXI- or FXII-directed strategies (Table 2).

**Table 2:** **Potential indications for factor (F) XII- or FXI-directed strategies**.

Indication	Rationale
Primary VTE prophylaxis	Long-acting strategies such as antisense oligonucleotides or antibodies permit simple and safe single-dose regimens for extended thromboprophylaxis in medically ill patients or after major orthopedic surgery
Extended VTE treatment	May be safer than current therapies for extended VTE treatment in patients with unprovoked or cancer-associated VTE
Prevention of recurrent ischemia after acute coronary syndrome in patients with or without atrial fibrillation	May provide a safer anticoagulant platform on top of single or dual antiplatelet therapy
End-stage renal disease	May be safe and effective for reducing cardiovascular death, myocardial infarction, and stroke in patients on hemodialysis
High-risk atrial fibrillation patients	May be safer than current therapies for stroke prevention in atrial fibrillation patients at high risk for bleeding such as those with a history of major bleeding or with end-stage renal disease
Medical devices	May be more effective and safer than current therapies to prevent clotting on mechanical heart valves, left ventricular assist devices, small caliber grafts, or central venous catheters
Extracorporeal circuits	May be more effective and safer than heparin to prevent clotting on extracorporeal membrane oxygenator or cardiopulmonary bypass circuits

*VTE, venous thromboembolism*.

## Potential Indications for FXII- or FXI-Directed Therapies

Until the question of whether FXII or FXI monotherapy is sufficient for treatment of established venous or arterial thrombosis is answered, it may be better to focus on prevention of arterial or venous thrombosis. FXII- or FXI-directed ASOs are best suited for chronic indications because of their slow onset of action. These might include prevention of cardiovascular events in patients with chronic kidney disease, and stroke prevention in atrial fibrillation patients at high risk for bleeding, such as those with end-stage renal disease who are on hemodialysis. Extended anticoagulation therapy in patients with unprovoked VTE is another potential indication because such patients have risk of recurrent thrombosis (~10% at 1 year and ~30% at 5 years) if anticoagulant therapy is stopped (51). Although many of them are maintained on DOACs, even when used at reduced doses, there is a risk of bleeding (52). FXII- or FXI-directed strategies may not only be safer, but adherence may also be better with once or twice monthly injections of ASOs or antibodies than with oral medications that must be taken once or twice daily. These possibilities need to be tested.

Stroke prevention in patients with atrial fibrillation and severe kidney disease represents an additional unmet medical need because the DOACs have not been tested in this setting, and because there is uncertainty as to whether the harms of warfarin outweigh its benefits. As to which is the better target for this indication, FXI overshadows FXII because FXI inhibition will prevent thrombus stabilization and growth regardless of whether the stimulus for clotting at sites of plaque disruption or in the left atrial appendage is driven by TF or by FXII activation by polyphosphates. Inhibition of FXI may also better attenuate clotting on the hemodialysis circuit, thereby obviating the need for heparin and further lowering the risk of bleeding. Even without atrial fibrillation, patients on hemodialysis are at risk of cardiovascular events and such events are responsible for at least 50% of the mortality. Therefore, a FXI-directed strategy may be beneficial to safely prevent such events in hemodialysis patients with or without atrial fibrillation.

Additional indications for patients requiring medical devices are evident. FXII- or FXI-directed therapies may be safer than heparin for prevention of clotting on extracorporeal membrane oxygenation circuits, and safer than warfarin for prevention of thromboembolic events in patients with left ventricular assist devices. In patients with mechanical heart valves (53), FXI-directed strategies may be very effective in this setting because FXI depletion abolished mechanical valve-induced thrombin generation *in vitro* (38). It is notable that dabigatran failed against warfarin (53); a finding that prompted black box warnings against the use of DOACs in such patients.

Factor XI-directed strategies may also provide a better alternative in acute coronary syndrome patients requiring anticoagulant therapy on top of single or dual antiplatelet therapy (Table 2). Thus, even though rivaroxaban reduced the risk of recurrent ischemic events and stent thrombosis when added to dual antiplatelet therapy in such patients, these beneficial effects came at a cost of increased bleeding, including intracranial bleeding (54). FXI-directed strategies are likely to be safer than rivaroxaban and should not only block contact activation on stents, but should also prevent FXI-mediated thrombus stabilization and growth. Thus, there are numerous potential indications for targeting FXI and FXII that require investigation.

## Conclusion and Future Directions

Recent advances in our understanding of the biochemistry of coagulation have revealed novel targets beyond those involved in the terminal reactions of the coagulation pathway. With evidence that the contact system is important for thrombus stabilization and growth, FXI and FXII have emerged as promising targets for new anticoagulants that may prove to be safer than those that inhibit FXa or thrombin. ASOs, antibodies, and small molecules are expanding the armamentarium of agents, and it will be necessary to determine whether FXI or FXII is the better target and to compare the efficacy and safety of these new strategies with current standards of care for prevention or treatment of thrombosis. The first priority should be selection of indications that focus on unmet medical needs, particularly those where current therapies are limited in both efficacy and safety. The clinical potential of FXII- and FXI-directed anticoagulant strategies represent an exciting new era in anticoagulation that should reduce the risk bleeding without compromising efficacy.

## Author Contributions

All the authors listed have made substantial, direct, and intellectual contribution to the work and approved it for publication.

## Conflict of Interest Statement

JF has no reported conflicts. JW has served as a consultant and has received honoraria from IONIS Pharmaceuticals, Janssen, Bayer, Boehringer Ingelheim, Bristol-Myers Squibb, Pfizer, Merck, and Daiichi Sankyo.
